# Evaluating Crowdsourced Data Collection for Carceral Death Surveillance: Pilot Study Using Amazon Mechanical Turk

**DOI:** 10.2196/79315

**Published:** 2026-05-15

**Authors:** Emily Wang, Julia Healey-Parera, Amy Duan, David H Cloud, Mike Dolan Fliss, Lauren Brinkley-Rubinstein

**Affiliations:** 1 Department of Population Health Sciences School of Medicine Duke University Durham, NC United States; 2 Injury Prevention Research Center University of North Carolina at Chapel Hill Chapel Hill, NC United States

**Keywords:** carceral health, deaths in custody, mortality surveillance, crowdsourcing, Amazon Mechanical Turk, structured data abstraction, digital epidemiology, health equity

## Abstract

**Background:**

People who are incarcerated face significantly higher health risks than the general population, yet deaths in custody remain underreported and poorly monitored by public health systems. Although the federal Death in Custody Reporting Act requires reporting of all deaths in correctional facilities to the US Department of Justice, reporting has been inconsistent, delayed, and often publicly inaccessible. Consequently, researchers have turned to press releases issued by correctional agencies as one of the few timely sources of information on deaths in custody. However, these press releases vary widely in content and structure, making standardized data extraction difficult. Crowdsourcing platforms such as Amazon Mechanical Turk (MTurk) may offer a faster, low-cost method for gathering data, but their utility in this setting remains untested.

**Objective:**

This pilot study evaluated whether MTurk could be used to extract structured information from press releases about deaths in custody.

**Methods:**

We selected 144 press releases describing deaths between 2000 and 2023 from state prison systems and Immigration and Customs Enforcement. Each press release was assigned to 3 MTurk crowd workers (who were required to be English speaking and located in the United States), resulting in 432 individual responses. Workers were informed in advance that the task involved reviewing sensitive content related to deaths in custody. Crowd workers completed a 16-question form aligned with Death in Custody Reporting Act variables, including age, race and ethnicity, date of death, and facility location. Data quality was assessed using strict concordance (all 3 responses matched), 2-way concordance (2 of 3 responses matched), and qualitative review of common errors. Task completion time was also recorded. Sampling included complete subsets of selected press releases and a stratified subset from systems with more complex reporting formats.

**Results:**

All 144 entries were completed within 48 hours. However, agreement across crowd workers was low: strict concordance was 14.2% (20/144) for age, 12.3% (18/144) for race or ethnicity, and 11.4% (16/144) for date of birth. Qualitative review identified frequent errors, missing data, and inattentive or automated responses. Crowd workers often misinterpreted system-specific terminology or copied placeholders instead of extracting information from the source. The low agreement indicated that this baseline MTurk configuration produced insufficient data quality for more resource-intensive use.

**Conclusions:**

MTurk enabled rapid task completion but produced low-quality results when applied to extracting structured data from carceral press releases. These findings suggest that general crowdsourcing platforms are poorly suited to complex data abstraction tasks without additional training or oversight. With improved task design or support from artificial intelligence tools, crowdsourcing may help address gaps in the surveillance of deaths in custody. Long-term improvements will require consistent, transparent, and standardized reporting practices across correctional institutions.

## Introduction

With more than 1.2 million people held in state and federal prisons, the United States operates the most expansive prison system in the world [1]. As mass incarceration has grown, premature deaths in custody have emerged as a mounting public health crisis, increasing in scale while receiving limited attention from public health systems [2,3]. People who are incarcerated experience substantial health disparities shaped by the conditions of confinement [4,5]. Overcrowding, idleness, unsanitary living spaces, inadequate nutrition, limited access to medical and mental health care, isolation, and exposure to violence all contribute to worsening health and increased risk of premature death [2-6]. Since 2001, the prison mortality rate has increased by 77%, with particularly steep rises in suicides and drug- and alcohol-related deaths. Between 2001 and 2018, drug- and alcohol-related deaths increased more than sixfold, and suicides rose by more than 85% [7,8].

Despite the growing number of deaths in US jails and prisons, the nation lacks a coordinated public health and policy infrastructure for tracking, reporting, and responding to carceral mortality [2,9]. The Death in Custody Reporting Act (DCRA) requires state and local agencies, as well as federal agencies within the Department of Justice, to report all deaths in custody to the Attorney General, including deaths occurring during arrest; in transit; and within correctional facilities such as jails, prisons, and juvenile detention centers. However, DCRA does not apply to agencies outside the Department of Justice, such as US Immigration and Customs Enforcement (ICE), which is housed within the Department of Homeland Security and governed instead by internal policies and congressional directives requiring separate public reporting [10-12].

This fragmented jurisdictional landscape contributes to persistent gaps, delays, and inconsistencies in mortality data, which undermine efforts to track trends, hold institutions accountable for conditions that contribute to preventable deaths in custody, and seek justice for those who die behind bars and their families [2,9]. A recent national study found that only 1 prison system, Iowa, publicly releases death data that are both timely and complete under DCRA standards. Over one-third of systems release no individual-level death data at all, while most others either publish information with substantial delays or omit essential details such as age, cause of death, or facility [11].

Although incarceration is a key structural determinant of health inequities in the United States [4], responsibility for tracking deaths in custody has largely remained outside the public health system. Surveillance of deaths in jails, prisons, and other detention facilities is not integrated into Centers for Disease Control and Prevention mortality systems, and carceral health research receives minimal support from the National Institutes of Health [13]. This lack of public health ownership is reflected in vital records systems, including death certificates, which do not consistently indicate whether a death occurred in custody. Although adding a standardized field or checkbox has been proposed, it has not been implemented nationally [2]. Because federal funding priorities shape state and local public health capacity, these gaps further constrain the ability of public health systems to monitor and respond to carceral mortality [2,11,14].

In 2019, federal responsibility for collecting deaths-in-custody data shifted from the Bureau of Justice Statistics, a statistical agency with a mandate for public reporting, to the Bureau of Justice Assistance, a grant-making agency with less experience in data collection and analysis. This transition raised concerns about data quality and transparency. Although Bureau of Justice Assistance began publishing carceral mortality dashboards in 2024, the datasets contain clear errors and omissions, including states listed as having no deaths and entries missing key information such as cause of death or demographic characteristics [14]. These shortcomings have further weakened confidence in federal oversight of deaths in custody.

Surveillance, transparency, and accountability are closely linked in the context of carceral mortality. Transparency depends on timely, public reporting of deaths by correctional systems, yet official data sources often fail to meet this standard [2,9]. National analyses of DCRA compliance show that 39% of prison systems release no individual-level death data. Among systems that do report deaths, only Iowa publishes complete and timely information that includes all DCRA-required elements within the federal 3-month reporting window [11]. Because official carceral mortality data are frequently delayed or incomplete, press releases issued by carceral systems often provide the earliest publicly available information about deaths in custody. However, both the availability and content of these press releases vary widely across jurisdictions.

This study builds on the work of the Third City Project at Duke University, which was launched during the COVID-19 pandemic to improve transparency in carceral health data. While initially focused on tracking infectious disease outbreaks in prisons and jails, the project later expanded to address broader gaps in mortality reporting. Through manual review and coding of hundreds of press releases, the research team found that although these documents often contain valuable information, extracting structured data from them is labor intensive and difficult to scale [11,14].

These constraints prompted consideration of alternative approaches that might support more timely data abstraction without relying exclusively on trained research staff. One such approach is crowdsourcing, which has been increasingly used in health and social science research to complete discrete, repeatable tasks at scale. Platforms such as Amazon Mechanical Turk (MTurk) and Prolific are commonly used to recruit participants for surveys and behavioral experiments, offering speed, relatively low cost, and access to large respondent pools [6,15-19]. In public health, crowdsourcing has been applied to studies of vaccine attitudes [20], substance use [21], and disability [22], as well as in criminology to research on public attitudes toward incarceration and punishment [23,24].

Beyond survey-based research, crowdsourcing has also been used for content-focused tasks in fields such as computer science, information science, and the digital humanities, including annotating scientific abstracts, labeling archival materials, and coding legal or biomedical texts [15-19,25,26]. In these applications, crowd workers extract or classify information from existing documents rather than generate original responses. Such studies typically rely on relatively controlled source materials with consistent formats, specialized vocabularies, and detailed task instructions or training.

By contrast, press releases reporting deaths in custody vary widely in structure, language, and level of detail; often include institution-specific terminology; and reflect differing norms around transparency and disclosure across carceral systems [11]. These features introduce challenges that may limit the effectiveness of general-purpose crowdsourcing approaches designed for more standardized materials.

At the same time, press releases are among the few mechanisms through which individual-level information about deaths in custody enters the public record within days or weeks of a death, despite substantial variation in content and completeness [11]. Consequently, they represent a constrained but potentially valuable data source for improving the timeliness of carceral mortality surveillance.

This pilot study examines the use of MTurk to extract structured mortality information from press releases describing deaths in custody, using a basic task design and minimal crowd worker screening or training to characterize baseline performance.

## Methods

### Study Design and Data Sources

As part of its ongoing work to improve transparency around carceral health outcomes, the Third City Project systematically collects and standardizes data on in-custody deaths reported through state prison system press releases [11,27]. Press releases were selected from a broader dataset of carceral mortality notifications compiled by the Third City Project, which includes public announcements from state Departments of Corrections; the Federal Bureau of Prisons; ICE; and select territories such as Washington, District of Columbia, and Puerto Rico.

For this pilot study, we analyzed 144 press releases from state prison systems and ICE. Press releases from jails, the Federal Bureau of Prisons, Washington, District of Columbia, and Puerto Rico were not included. Consequently, the MTurk sample represents deaths occurring in state prisons and immigration detention but not in jails or federal prisons.

Press releases were sourced from official Department of Corrections websites, agency social media accounts, and periodic bulletins. Relevant information was manually extracted and entered into standardized, state-specific Qualtrics (Qualtrics Inc) forms capturing variables such as the decedent’s name, demographic characteristics, date and location of death, cause of death, facility name, and incarceration date. In some cases, states also maintained dashboards or published aggregate mortality statistics. The dataset was cleaned and standardized using custom R scripts prior to analysis.

### Crowdsourcing Platform and Procedure

MTurk is a web-based crowdsourcing platform that allows employers, known as requesters, to post microtasks online—called human intelligence tasks (HITs)—for human workers, or “turkers,” to complete in exchange for compensation. Each HIT is a self-contained assignment, such as identifying content in a photo, extracting information from a document, or answering survey questions. Once an HIT is completed, the requester reviews the quality of the submission and determines whether the work meets standards for payment.

In July 2024, we conducted a pilot study using MTurk to evaluate whether crowd workers could extract structured data from publicly available prison death press releases. Each HIT required workers to complete a structured 16-question form based on a single press release. For each entry, workers were provided the decedent’s name, a direct link to the press release, the correctional system involved, and system-specific instructions. Variables aligned with DCRA requirements and included age, race or ethnicity, date and location of death, cause of death, and facility name. Workers were instructed to leave fields blank if information was not reported.

Each press release was independently assigned to 3 crowd workers to enable comparison across responses. Task instructions were intentionally brief, and no formal attention checks were embedded, to observe baseline performance under low-barrier conditions. Because ICE press releases often include more detailed immigration and medical histories, a supplemental survey containing 8 additional questions was deployed for ICE entries only. Crowd workers were informed prior to task acceptance that the assignment involved reviewing a press release describing a death in custody and could decline participation without penalty.

### Sampling

Entries were selected by taking a complete sample of press releases published in 2023 from the larger Third City dataset (n=7184 as of August 2024), supplemented by a stratified subsample of up to 5 press releases from each of 5 systems with more complex reporting formats: Arkansas, Florida, Montana, Texas, and ICE.

Because this pilot was designed to assess feasibility rather than generate population-level estimates, we did not aim for a balanced or representative sample. Instead, we deliberately oversampled systems with more complex reporting structures to ensure inclusion of both straightforward and challenging cases. This design should be interpreted as a feasibility test rather than as representative of typical state prison press releases.

### Data Quality Assessment and Statistical Analysis

To assess data quality, we conducted a concordance analysis comparing responses submitted by the 3 crowd workers assigned to each press release. Analyses focused on variables required under the DCRA, as these fields are central to carceral mortality surveillance.

We calculated the percentage of entries in which all 3 crowd workers provided the same response (strict concordance) and the percentage in which 2 of 3 responses matched (2-way concordance). These measures reflect complete and majority agreement, respectively, and exclude partial matches. CIs were calculated for all concordance estimates. Discrepancies were reviewed qualitatively to identify common sources of error, including missing or skipped fields, inconsistent interpretation of press release content, and copying of placeholder examples rather than source material.

To limit risks of misclassification, MTurk outputs were treated strictly as data extraction measures rather than definitive classifications of press release content. Crowd worker responses were compared to data manually extracted by Third City Project research assistants as a reference point for identifying discrepancies, rather than as a formal gold standard validation. Assigning multiple independent crowd workers to each press release allowed us to quantify uncertainty and identify entries with high disagreement. Variables were defined using language commonly used in press releases (eg, “facility housed” and “press release date”) to avoid binary or morally loaded classifications.

### Ethical Considerations

This study involved analysis of publicly available press releases and was reviewed and approved by the Duke University Institutional Review Board (Pro00111898). All source materials were published by correctional agencies and accessed through official public channels. No private or nonpublic records were used.

Decedent names and links to press releases were retained because they were part of the original public disclosures and were necessary to assess whether identifying information was consistently reported and could be reliably extracted across systems. No additional personal information was collected, inferred, or linked beyond what was included in the publicly released materials.

Crowd workers were informed prior to task acceptance that the assignment involved reviewing a press release describing a death in custody. This warning appeared in the task title and description on the MTurk marketplace, allowing workers to opt out without penalty if they did not wish to engage with the content. All press releases were reviewed in advance by Third City Project research assistants to screen for graphic or disturbing material before inclusion in the task.

Compensation was set based on task length and complexity and benchmarked against prevailing MTurk rates to ensure payment above the platform average for comparable tasks. The study did not require workers to generate original content or make judgments about responsibility or cause beyond extracting information explicitly stated in the source material.

To limit the risk of misclassification that could affect public narratives about deaths in custody, MTurk outputs were treated as data extraction rather than definitive classifications. Each press release was reviewed by multiple independent crowd workers, and disagreement across responses was used to identify uncertainty rather than to assert accuracy. Results were reported in aggregate, and high-disagreement cases were flagged for review rather than interpreted as definitive records.

## Results

The MTurk pilot demonstrated that while crowdsourcing offers a rapid turnaround for data collection, it yielded low-quality results when applied to carceral death press releases. All 144 assigned tasks were completed within 2 days, reflecting the efficiency of the platform. However, concordance analysis revealed low consistency across crowd worker responses.

Table 1 presents 2-way (2-of-3) concordance for variables aligned with DCRA reporting requirements, along with 95% CIs. Two-way concordance captures how often independently submitted responses converged on the same value for a given field.

**Table 1:** Two-way concordance of Amazon Mechanical Turk crowd worker responses for variables extracted from prison mortality press releases.

Variable	Two-way concordance (95% CI)
First name	0.7092 (0.6268-0.7826)
Last name	0.6596 (0.5751-0.7372)
Race or ethnicity	0.6738 (0.5898-0.7503)
Age	0.5532 (0.4672-0.6369)
Gender	0.5957 (0.5099-0.6775)
Location of death	0.3688 (0.2892-0.4541)
Date of death	0.4255 (0.3427-0.5115)
Cause of death	0.3050 (0.2303-0.3880)
Facility housed	0.5035 (0.4182-0.5888)
Press release date	0.4751 (0.3905-0.5609)

Two-way concordance was highest for basic identifying information. Matching responses were most common for first name (0.7092, 95% CI 0.6268-0.7826), last name (0.6596, 95% CI 0.5751-0.7372), and race or ethnicity (0.6738, 95% CI 0.5898-0.7503). Moderate levels of concordance were observed for age (0.5532, 95% CI 0.4672-0.6369), gender (0.5957, 95% CI 0.5099-0.6775), and facility housed (0.5035, 95% CI 0.4182-0.5888).

Lower 2-way concordance was observed for variables that were inconsistently reported or embedded within narrative text. These included date of death (0.4255, 95% CI 0.3427-0.5115), press release date (0.4751, 95% CI 0.3905-0.5609), location of death (0.3688, 95% CI 0.2892-0.4541), and cause of death (0.3050, 95% CI 0.2303-0.3880). Overall, convergence across independent responses was higher for discrete, clearly labeled fields than for information requiring interpretation across multiple sections of a press release.

Table 2 presents strict 3-way concordance, defined as identical responses across all 3 independent crowd workers for a given field. This measure represents a conservative benchmark of consistency and reflects the upper bound of agreement achievable without review or correction.

**Table 2:** Three-way concordance of Amazon Mechanical Turk crowd worker responses for variables extracted from prison mortality press releases.

Variable	Three-way concordance (95% CI)
First name	0.2553 (0.1857-0.3355)
Last name	0.2340 (0.1669-0.3127)
Race or ethnicity	0.1277 (0.0774-0.1942)
Age	0.1418 (0.0888-0.2105)
Gender	0.0639 (0.0296-0.1177)
Location of death	0.0567 (0.0248-0.1087)
Date of death	0.2553 (0.0345-0.1266)
Cause of death	0.0425 (0.0158-0.0903)
Facility housed	0.1277 (0.0774-0.1942)
Press release date	0.1206 (0.0718-0.1860)

For demographic and contextual fields, 3-way concordance was substantially lower. Concordance for age (0.1418, 95% CI 0.0888-0.2105), race or ethnicity (0.1277, 95% CI 0.0774-0.1942), and facility housed (0.1277, 95% CI 0.0774-0.1942) indicated limited consistency across independently submitted responses. The lowest levels of 3-way concordance were observed for cause of death (0.0425, 95% CI 0.0158-0.0903), location of death (0.0567, 95% CI 0.0248-0.1087), and gender (0.0639, 95% CI 0.0296-0.1177), all of which were frequently missing, inconsistently reported, or embedded within narrative text.

Qualitative review identified several common sources of error. First, some crowd workers submitted inaccurate data across multiple fields. For instance, several age and race entries appeared to be inferred from photographs in the press release rather than stated information. Second, crowd workers often skipped fields that were present in the source or copied placeholder examples instead of completing the form. Finally, for systems with more complex reporting formats, such as ICE or Texas, crowd workers sometimes returned overly specific but incorrect facility names, suggesting reliance on external sources or automated tools rather than the press release itself.

## Discussion

This study assessed whether MTurk could serve as a scalable tool to support the timely collection of carceral mortality data, a need made urgent by persistent delays, data gaps, and inconsistent reporting across carceral systems [2,10,11]. Although crowdsourcing platforms are widely used in public health research for their speed and low cost, their performance in complex data abstraction tasks is less well understood. Therefore, we conducted a feasibility study using a deliberately low-barrier task design, with minimal instructions and no enhanced crowd worker screening or training, to characterize baseline performance under conditions that might be considered for rapid, low-cost deployment. Under these conditions, MTurk enabled rapid task completion but produced low concordance across key mortality fields.

Crowdsourcing has gained traction in public health for tasks such as disease surveillance, participatory epidemiology, and outbreak monitoring due to its capacity to generate large volumes of timely data at low cost [17-22,25,26,28,29]. While their speed and scalability are appealing, researchers have raised concerns about data quality, sampling bias, and crowd worker attentiveness, especially in studies requiring accurate or complex data, where these tools may introduce significant limitations [25,26,28]. Our findings are consistent with these concerns.

Across many variables required under the DCRA, concordance between independently submitted crowd worker responses was low. Even for core DCRA fields such as age, race or ethnicity, and date of birth, strict 3-way agreement occurred in fewer than 15% (20/144, 18/144, and 37/144, respectively) of cases. These results indicate substantial inconsistency in how crowd workers interpreted and extracted information from the same press releases and suggest that baseline MTurk configurations are poorly suited for unreviewed abstraction of mortality data intended to support surveillance or accountability.

One factor contributing to these limitations appears to be the specialized nature of carceral press releases. These documents often contain system-specific terminology, unclear abbreviations, and facility naming conventions that may be unfamiliar to general audiences. Crowd workers without prior experience or training appeared to struggle with these features, leading to errors even when the correct information was present. Errors were particularly common for variables that are central to DCRA reporting but inconsistently labeled in press releases, such as cause and location of death. In some cases, responses were overly specific but incorrect, suggesting reliance on external searches or automated tools rather than the press release itself.

Despite these limitations, MTurk may have limited utility if applied in more constrained or carefully designed ways. More selective worker eligibility criteria, such as higher approval thresholds or prior experience with similar tasks, could improve performance. Providing clearer field-level guidance or brief tutorials tailored to carceral terminology may also reduce misinterpretation. For complex or sensitive variables, hybrid approaches that combine crowdsourcing with trained review may be necessary [19,28].

Alternative platforms such as Prolific may offer stronger infrastructure for academic research. Prolific provides tools for prescreening, attention checks, and improved compensation, which have been associated with higher-quality data in survey-based studies. Some evidence suggests that experienced crowd workers may be migrating away from MTurk in favor of platforms with better pay and task design [16]. Past public health studies have successfully used Prolific to conduct psychometrically oriented substance use research and administer surveys to determine perceptions of COVID-19 among the public [25-27]. However, it remains unclear whether these advantages would translate to complex data abstraction tasks involving heterogeneous and institutionally specific source materials, such as carceral death press releases.

Emerging applications of large language models (LLMs) also merit consideration. LLMs are increasingly used to extract information from unstructured text and may offer potential for processing press releases and other narrative data related to carceral deaths [30,31]. Exploring the use of LLMs for carceral mortality surveillance may be a worthwhile direction for future research.

Our pilot used a low-barrier approach to assess baseline feasibility for a specific research objective to identify limitations and guide future applications of this method. Several strategies may improve the reliability of future efforts to extract structured data from press releases reporting deaths in custody. Dividing the task into smaller components may help crowd workers navigate complex narratives more effectively. Providing field-specific examples and a brief tutorial could improve clarity and reduce misinterpretation. Limiting participation to crowd workers with higher approval ratings or prior experience in similar tasks may also enhance consistency [25,32].

Several limitations should be considered when interpreting these findings. First, the analysis relied on a single crowdsourcing platform and reflects the performance of its general worker pool without enhanced screening or training. Second, the pilot included a relatively small sample of 144 entries and intentionally oversampled systems with more complex reporting formats. As a result, the findings describe this specific feasibility test and should not be generalized to all state prison press releases. Third, the source materials themselves varied widely in structure and completeness, with essential information often missing or ambiguously reported. Fourth, although concordance was assessed across multiple workers, we did not validate MTurk outputs against a gold standard dataset coded by trained experts. Future studies should directly compare crowdsourced extraction to expert abstraction to better assess accuracy. Finally, this pilot was not designed to evaluate how task-level factors, such as compensation, task length, or worker background, affected performance. Examining these factors is an important direction for future work.

Crowdsourcing methods also raise ethical concerns. These platforms often rely on low-paid labor to perform tasks requiring sustained attention and judgment. Asking untrained workers to process sensitive information about deaths in custody raises concerns about fairness, emotional burden, and responsibility in digital labor. In addition, this approach carries a risk of misclassifying carceral death data, which can have serious consequences. Records of deaths in custody carry legal, political, and public health significance, and inaccuracies may have unintended downstream effects.

As deaths in custody remain an urgent but undermonitored public health concern, there is growing interest in approaches that could improve the timeliness of mortality data consistent with DCRA requirements. In this feasibility pilot, MTurk enabled rapid task completion but produced data with limited reliability for extracting several key mortality variables. These findings reflect the specific low-barrier task design and conditions tested here and indicate that caution is warranted when applying general-purpose crowdsourcing platforms to carceral mortality surveillance. Future research should examine whether more targeted task design, alternative platforms, or complementary automated approaches can improve performance, alongside continued efforts to strengthen standardized and transparent public reporting of deaths in custody.

## Abbreviations

DCRA: Death in Custody Reporting Act
HIT: human intelligence task
ICE: Immigration and Customs Enforcement
LLM: large language model
MTurk: Amazon Mechanical Turk
